# Epigenome-Wide Search for Distinctive Methylation Biomarkers of Endothelial and Leukocyte DNA

**DOI:** 10.3390/epigenomes9040053

**Published:** 2025-12-17

**Authors:** Valeria A. Korolenya, Maxim L. Filipenko, Mariya A. Smetanina

**Affiliations:** 1 Laboratory of Pharmacogenomics, Institute of Chemical Biology and Fundamental Medicine (ICBFM) SB RAS, Novosibirsk 630090, Russia; 2 Department of Natural Sciences, Novosibirsk State University (NSU), Novosibirsk 630090, Russia; 3 Department of Fundamental Medicine, Institute of Medicine and Medical Technologies, Novosibirsk State University (NSU), Novosibirsk 630090, Russia

**Keywords:** DNA methylation, endothelial cells, leukocytes, microarray analysis

## Abstract

The endothelium, as the inner layer of the vascular wall, is in constant contact with blood components, so that leukocytes have the ability to adhere to endotheliocytes and penetrate to the subendothelial space. When studying heterogenic vascular samples containing endothelial cells or pathological processes related to inflammation within the endothelium, it may be necessary to distinguish DNA by endothelial and leukocyte origin, which is possible due to its specific epigenetic modifications. To identify CpG loci that could serve as markers for endothelial cells, we searched for their distinctive stable methylated or demethylated states by applying marginal filtering (selecting CpG loci with methylation Beta values closer to 0 and 1) to the microarray data and identified 47 CpG loci with relatively stable methylation/demethylation status that differentiate endothelial (HUVEC, HCMEC, HPAEC, HPMEC, and LSEC) DNA from leukocyte (granulocytes, monocytes, and lymphocytes) DNA. In addition, we compared CpG loci with high and low levels of DNA methylation between different types of endothelial cells and leukocytes. We believe that the obtained data will hopefully facilitate further studies on endothelial dysfunction.

## 1. Introduction

The inner lining of blood vessels is represented by a layer of endothelial cells (ECs), which act as a selective barrier and regulate vascular tone, hemostasis, angiogenesis, and inflammation [1]. Disruption of these cells can be linked with endothelial dysfunction associated with diseases such as hypertension, diabetes, atherosclerosis, coronary heart disease, peripheral arterial disease, myocardial infarction, hyperglycemia, renal failure, Alzheimer’s disease, and chronic venous disease [2,3,4]; ECs have been shown to have tissue-specific patterns of gene expression, indicating their heterogeneity [5]. ECs facing the blood flow are constantly in contact with leukocytes (LCs) that interact with them by attaching, rolling, and migrating into the vessel wall [6]. Although LCs make up only 1% of the blood, they are among the most active nucleated cells [7]. Neutrophils are the most abundant white blood cells: 40–60% of the absolute number of LCs, with lymphocytes (T, B cells, natural killer (NK) cells) accounting for 20 to 40%. Monocytes are much less common (2–8%), and the least numerous are eosinophils (0–4%) and basophils (0.5–1%), which, like neutrophils, are granulocytes [8].

To determine the origin of DNA, the following epigenetic markers are being used: cytosine methylation, fragmentation pattern, and fragment topology [9]. DNA methylation is one of the mechanisms regulating gene expression by altering the degree of binding of transcription factors to regulatory regions [10,11], as well as the formation of heterochromatin [12,13]. The process of EC differentiation is associated with DNA methylation [14,15], regulating the expression of EC-specific genes, which has also been demonstrated during the induction of pluripotency in human umbilical vein ECs (HUVEC) [16]; after the differentiation of ECs is completed, they acquire stable and inherited DNA methylation marks retained throughout EC division and aging.

DNA methylation profiling is performed using chemical conversion of unmethylated cytosines with bisulfite, antibodies, or methylation-specific enzymes, followed by methylation-specific PCR, combined bisulfite-restriction analysis, bisulfite-free sequencing, or microarray analysis [17]. Microarray analysis is a high-throughput and reliable approach based on the complementary binding of nucleic acid fragments to thousands of oligonucleotides immobilized on a support, and its fluorescent signal is read by a machine [18]. Thus, the aim of this work was to search for endothelium-specific DNA methylation markers by analyzing available microarray data performed on ECs of different organ specificities and different types of LCs. For this, we compared HUVEC, human cardiac microvascular (HCMEC), human pulmonary arterial (HPAEC), human pulmonary microvascular (HPMEC) and liver sinusoidal (LSEC) ECs with LCs.

## 2. Results

### 2.1. Distinctive Features of DNA Methylation Within EC and LC Groups

**Figure 1:**
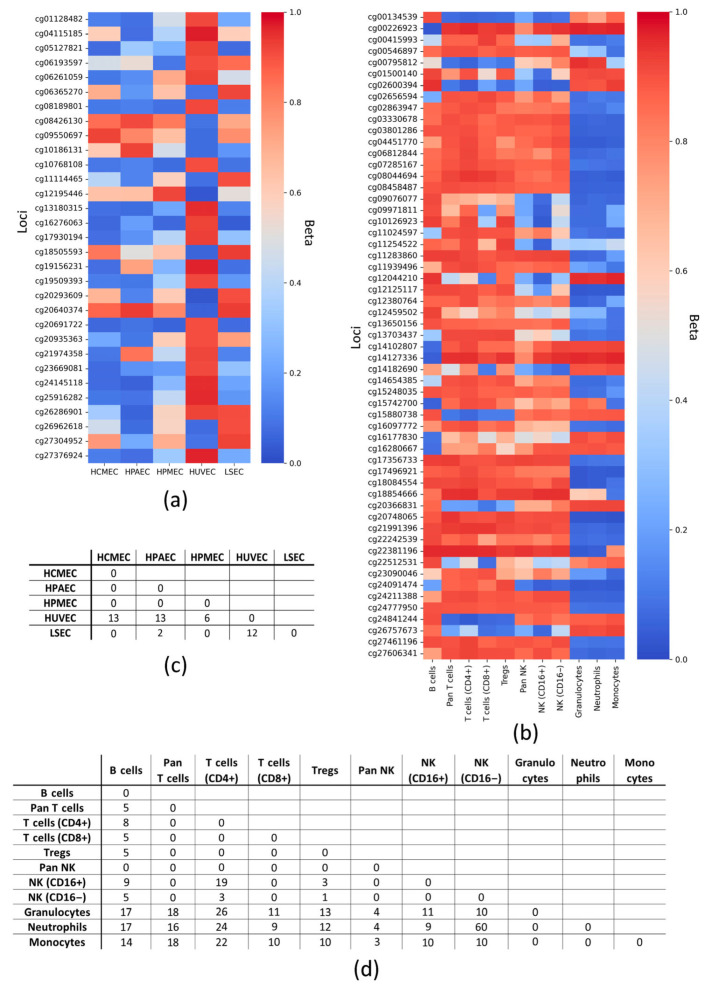
Differences in methylation of DNA loci with marginal Beta (Beta < 0.1 and Beta > 0.9) within cell groups. Heatmaps of marginal methylation patterns for all types of (**a**) endotheliocytes and (**b**) leukocytes. The graphs were constructed so that at least two cell subtypes had a polarly methylated locus, while the remaining types could have Beta outside the boundaries. Number of CpG loci with polar marginal Betas for each pair of cell subtypes within the groups: (**c**) endotheliocytes and (**d**) leukocytes. HCMEC—human cardiac microvascular endothelial cells; HPAEC—human pulmonary artery endothelial cells; HPMEC—human pulmonary microvascular endothelial cells; HUVEC—human umbilical vein endothelial cells; LSEC—liver sinusoidal endothelial cell; NK—natural killer cells; Tregs—regulatory T cells; CD—cluster of differentiation; Beta—average value of the proportion of methylated cytosines for a particular locus in a DNA sample; all loci are designated according to the Illumina identifier.

First, we compared CpG loci with marginal Beta (Beta < 0.1 and Beta > 0.9) (see Section 4) between cell types to identify epigenetic differences within the groups. To do this, we selected CpG loci that differed marginally between at least two cell types within a group. Thus, we obtained lists of 31 and 57 CpG loci that determine DNA methylation patterns within the studied ECs and LCs, respectively (Figure 1a,b, Supplementary File S1). In ECs, CpG loci identified as distinctive were associated with 18 genes (Table A1). Thus, 55 genes were identified in leukocytes (Table A2), and their functions reflect the features of those for immune cells (Supplementary Figure S1): response to stimuli, locomotion, cell activation, leukocyte differentiation, cell adhesion, cytokine production, phagocytosis, and interferon production.

As shown in Figure 1c, HUVEC stand out as the most prominent: 31 CpG loci distinguish them from the other cell types studied, so that cg20691722 is uniquely demethylated; while demethylated cg10768108 and cg16276063 distinguish HUVEC from HCMEC, HPMEC, and LSEC. Notably, all of the above loci are associated with a single gene, *SOX2OT* (Supplementary File S2). Calculation of the number of polar marginal loci at less stringent cutoffs (Beta < 0.2 and Beta > 0.8) reveals that LSEC and HPAEC are also quite distinct (Supplementary Table S1). The most frequent polar marginal loci (Beta < 0.1 and Beta > 0.9) in LCs are cg17356733 (*IFNGR2*), cg18084554 (*ARID3A*), cg20748065 (*POR*), cg21991396 (*NLRP3*), cg22381196 (*DHODH*), and cg27606341 (*FYB*) (Figure 1d, Supplementary File S2), and neutrophils and monocytes are the most prominent, although they do not differ significantly from each other according to the data analyzed (Supplementary Table S2).

Thus, the lists of CpG loci distinguishing each cell type within the groups (ECs and LCs) were identified.

### 2.2. Potential Markers of EC/LC DNA

**Figure 2:**
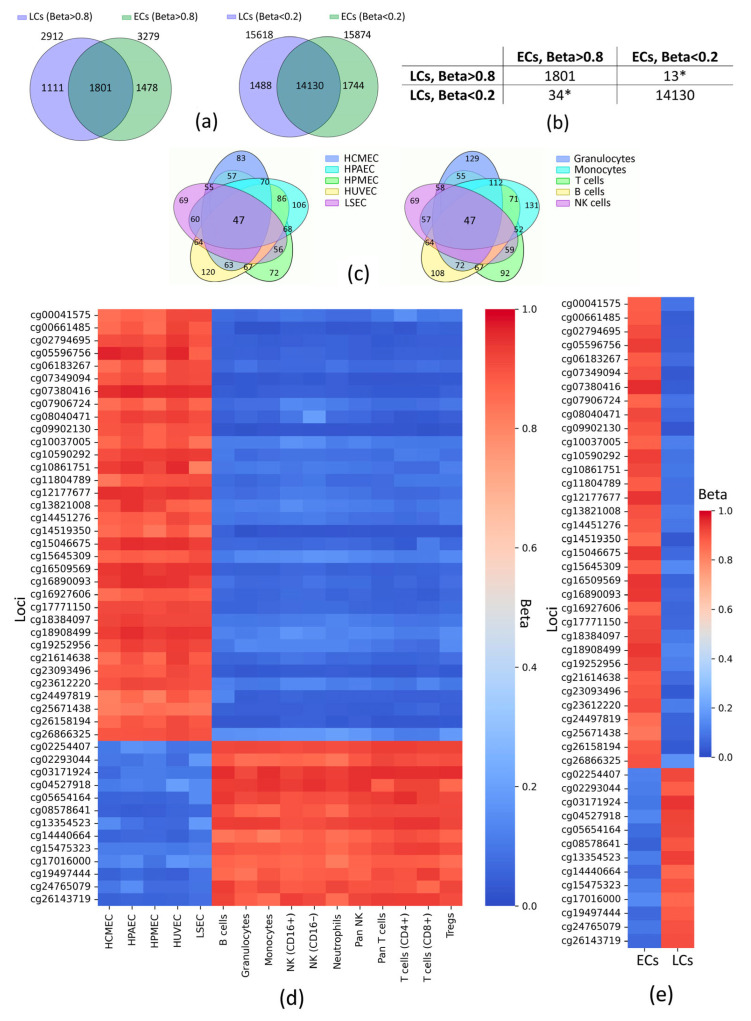
Methylation patterns between ECs and LCs (Beta < 0.2 and Beta > 0.8). (**a**) Venn diagrams showing the intersection of loci with similar marginal Beta. (**b**) The number of CpG loci included in the overlapping by marginal Betas. (**c**) Venn diagrams showing the intersections of loci that distinguish endothelial DNA from leukocyte DNA and vice versa (numbers in non-overlapping regions show the number of CpG loci identified for a particular cell subtype, numbers in overlapping regions show the number of CpG loci common to pairs of cell subtypes; some leukocytes are grouped into higher-order groups). Heatmaps of the loci distinguishing endothelial DNA from leukocyte DNA: (**d**) average Betas for all cell subtypes, (**e**) average Betas for each cell group. HCMEC—human cardiac microvascular endothelial cells; HPAEC—human pulmonary artery endothelial cells; HPMEC—human pulmonary microvascular endothelial cells; HUVEC—human umbilical vein endothelial cells; LSEC—liver sinusoidal endothelial cell; NK—natural killer cells; Tregs—regulatory T cells; CD—cluster of differentiation; ECs—endothelial cells; LCs—leukocytes; Beta—average value of the proportion of methylated cytosines of a particular locus in a DNA sample; * the sum of these values represents the number of polar marginal Betas between ECs and LCs; loci are designated according to the Illumina identifier.

Pairwise comparisons between ECs and LCs revealed numerous polar marginal CpG loci (Supplementary Table S3), but for a more generalized analysis, we searched for CpG loci that were similarly marginal for all cell types between the groups (Figure 2a,b) and polar between the groups (Figure 2c). Thus, we identified 47 CpG loci at Beta < 0.2 and Beta > 0.8 cutoffs, associated with 49 genes (Figure 2d,e, Supplementary File S3).

The identified 47 loci were associated with 49 genes related to biological processes that distinguish ECs from LCs (Table 1, Supplementary File S3). Thus, developmentally related genes have been identified (*AFF3*, *DNAI1*, *FOXI1*, *OSM*, *TG*). The unique functions of the studied cell groups suggest possible differences in the regulation of genes associated with metabolism (*AOAH*, *C1QTNF6*, *CD37*, *CHRNA6*, *DNAI1*, *HEM1*, *PSMB8*, *SLC67A1*, *TG*) and intracellular signaling (*ACAP1*, *CLEC1A*, *DOCK8*, *LCP1*, *RGS1*, *RIN2*, *SLA*, *UCN*, *CHRNA6*, *TG*). In fact, leukocytes differ from endothelial cells in their ability to move, which is typical for the identified genes associated with locomotion (*BIN2*, *DNAI1*, *DOCK8*, *LCP1*, *LSP1*). Genes responsible for the immune response (*AOAH*, *BATF*, *CYBC1*, *CD6*, *CST7*, *DOCK8*, *GCSAML*, *LCP1*, *OSM*, *PDCD1LG2*, *PSMB8*, *RGS1*, *TNFAIP8L2*, *TAP1*) and intercellular interactions (*BIN2*, *CD6*, *CDH1*, *CHRNA6*, *CLEC1A*, *CYTIP*, *DAPP1*, *DOCK8*, *OSM*, *TSPAN32*) were also identified.

One can see that 20 out of 47 (almost half) distinctive CpGs identified are located in TSS (promoter regions)—regulatory genomic regions—which defines their essential role in transcriptional regulation and, therefore, in cell phenotype determination. Only 14 out of 47 identified loci are located within the gene body. In LCs, 5 out of 13 stably methylated loci are located in CpG islands, while for ECs, this is only 1 out of 34. Thus, the set of polar marginal CpG loci that distinguish EC DNA from LC DNA was identified.

## 3. Discussion

A unique DNA methylation status for a particular cell type or tissue can be a marker associated with unique tissue-specific characteristics. When conducting large-scale DNA methylation analyses, profiles that describe the epigenetic features of the samples under study are usually generated, with the subsequent possibility of combining them with other omics data [19,20]. In the case of analyzing numerous DNA molecules isolated from tissues or cell cultures, such a probabilistic profile is essential for their characterization. Hypothetically, when determining the origin of a DNA molecule, using the entire profile is problematic because a specific locus on this molecule is either methylated or not. Therefore, it is reasonable to select boundary values at which the probability that a DNA locus from a particular cell is methylated/demethylated is maximized [21]. Therefore, we believe that such an approach could be very useful for searching for reliable marker loci.

To identify CpG loci with stable endothelium-specific methylation, we used the methylation profile of LCs as a background. First, we searched for marginal differences in methylation within each group (ECs and LCs). Notably, among the differentially methylated genes in ECs, the homeobox genes *HOXB7*, *HOXD3*, *HOXD4*, *HOXB5*, and *LHX6* clearly stood out (Table A1). These genes encode a family of transcription factors containing a helix-turn-helix homeobox (60 amino acids) and playing a key role in the processes of development, differentiation, and regulation of proliferation [22,23,24]. The *HOXB-AS3* gene was also detected; its long noncoding RNA encodes a peptide that suppresses smoking-induced inflammation in the bronchi, inhibits cell proliferation, and enhances apoptosis [25]. It can be noted that for HUVECs, all loci identified in pairwise comparisons within the EC group had relatively stable methylation, which may be associated with the unique features of these cells and provides a relatively high number of distinctive CpGs, compared to all other ECs (Figure 1b). A marker that distinguishes HUVEC from all other ECs studied was identified: the demethylated locus cg20691722, which is associated with the *SOX2OT* gene encoding a long noncoding RNA; this transcript is involved in embryogenesis, proliferation, and oncogenesis [26,27]. Notably, the loci predominantly demethylated in HUVEC and methylated in HCMEC, HPMEC, and LSEC (cg10768108, cg16276063) are also associated with the *SOX2OT* gene, emphasizing the role of this gene in the unique properties of HUVEC (e.g., higher proliferative activity). The cg20691722, cg10768108, and cg16276063 loci are located in relative proximity (within 12080 bp region) to each other on the same strand of chromosome 3 (Supplementary File S1).

The most common distinctive CpG loci for LCs were cg17356733, cg18084554, cg20748065, cg21991396, cg22381196, and cg27606341, associated with the *IFNGR2*, *ARID3A*, *POR*, *NLRP3*, *DHODH*, and *FYB* genes, respectively (Supplementary File S2). As shown in Supplementary Table S4, these robust methylation loci clearly distinguish lymphocytes from other LCs, which is likely due to different progenitors (lymphoid or myeloid) and/or maturation processes [28]. Almost all of the listed genes were related to immune regulation. Compared to myeloid cells, *IFNGR2* (interferon-gamma receptor 2) has a reduced expression level in lymphocytes [29], which may be associated with stable methylation of the locus related to this gene. The transcription factor ARID3a (AT-rich interaction domain 3A), which is involved in the production of interferon-alpha in neutrophils, is also associated with cytokines of inner immunity [30]. NLRP3 is a sensor component of the inflammasome, initiating an inflammatory response due to the release of IL-1β and IL-18 by caspase-1 [31]. Its gene (*NLRP3*) increased expression in leukocytes has been shown during atrial fibrillation [32]; its role in changing the phenotype of macrophages has been demonstrated, while its role in granulocytes remains poorly studied. NLRP3 can regulate T cell subsets, promoting the Th2 profile, and also affects the signature of IL-4, IL-13, IL1R1, ICOS, and MAF; meanwhile, B cells express low amounts of NLRP3 [33]. The product of another gene, *FYB*, is involved in T cell receptor signaling, promoting their activation and cytokine secretion [34]; its deficiency enhances the cytotoxicity of CD8+ T cells [35], and its high expression is an unfavorable marker of T cell lymphoma [36]. Conversely, increased *DHODH* expression is associated with the cancerous status of T cells in leukemia; decreased activity of its protein leads to decreased proliferation of cancer cells [37,38]. Of all the listed genes, the *POR* encoding cytochrome p450 oxidoreductase stands out: mutations in this gene lead to impaired sexual development and drug metabolism [39].

To search for distinguishing markers for ECs/LCs, we chose less stringent boundaries in order to obtain a more extended list of CpGs with polarly marginal Beta between the groups. As it is shown in Figure 2a, the number of CpGs with marginal Beta among the 25,946 loci studied is approximately the same: 19,153 for ECs and 18,530 for LCs; however, the number of loci with Beta < 0.2 is approximately 5 times higher than that with Beta > 0.8 in both groups. It is known that most of CpGs (up to 80%) in the mammalian genome are methylated, while CpG islands are often unmethylated [40]. Interestingly, about 73% of the loci studied were located in CpG islands: ~62% among CpGs with marginal Beta for ECs (~58% at Beta < 0.2, ~3% at Beta > 0.8) and ~59% of those—for LCs (~55% at Beta < 0.2, ~4% at Beta > 0.8) (Supplementary File S4). Overlapping CpGs were 55% and 62% of loci with Beta > 0.8 for ECs and LCs, respectively; whereas about 90% of loci with Beta < 0.2 were the same for both groups (Figure 2a). This may indicate that more similarity between ECs and LCs is observed at marginally low methylated CpG loci, which is somewhat contrary to the data showing that the presence of methylated cytosine is a stable epigenetic mark in various human cell types [41]; however, it is worth noting that our study is limited by principle of how microarrays are designed, so we do not expect our results to converge with the sequencing data. In fact, a limitation of our study is the determined set of loci examined, which is a consequence of using microarray data and makes it impossible to examine methylation sites across the entire genome. However, we believe that using data from microarray platforms of one manufacturer (Illumina), which handles both sample preparation and raw data processing, is an advantage of our study.

Ultimately, in spite of the aforementioned, in this study, we were able to detect potential epigenetic markers of ECs. Given the boundaries (Beta < 0.2 and Beta > 0.8), 47 CpG loci with polarly marginal Beta values were identified in ECs and LCs. As illustrated in Figure 2c,d,e, there were no outstanding values among the numbers of loci in pairwise intersections in both cell type groups. It is noteworthy that 72% (34 CpGs) of the identified loci were marginally low-methylated for LCs (see Table 1), which may be due to the fact that many of these loci-associated genes determine the phenotype of white blood cells (such as immune response and movement), and DNA methylation outside the gene body more often results in decreased gene activity [42]. This is very true for our data since a considerable number among those 34 CpGs belong to genes related to the immune response, such as *PSMB8*, *TAP1*, *RGS1*, *SELPLG*, and *TNFAIP8L2*, which reflects functional features of LCs in terms of Gene Ontology. It is equivalent to those 72% (34 CpGs) that were highly methylated for ECs. Within this number (72%), only 38% (13 out of 34) of CpGs are located in TSS (promoter regions) and 1 of 34—in CpG island, which is quite sparse in comparison to high-methylated for LCs 28% (13 CpGs) that are more densely enriched in TSS (7 out of 13 = 54%) and CpGs located within CpG islands (5 out of 13). Concerning other genomic elements, only 1 CpG locus in ECs and 1 CpG locus in LCs were located in enhancers.

Thus, using microarray data, we discovered the CpG loci that can serve as epigenetic markers for particular cells within the EC and LC groups, as well as for all ECs and LCs, which may aid in studying the epigenetics of endothelial dysfunction.

## 4. Materials and Methods

**Figure 3:**
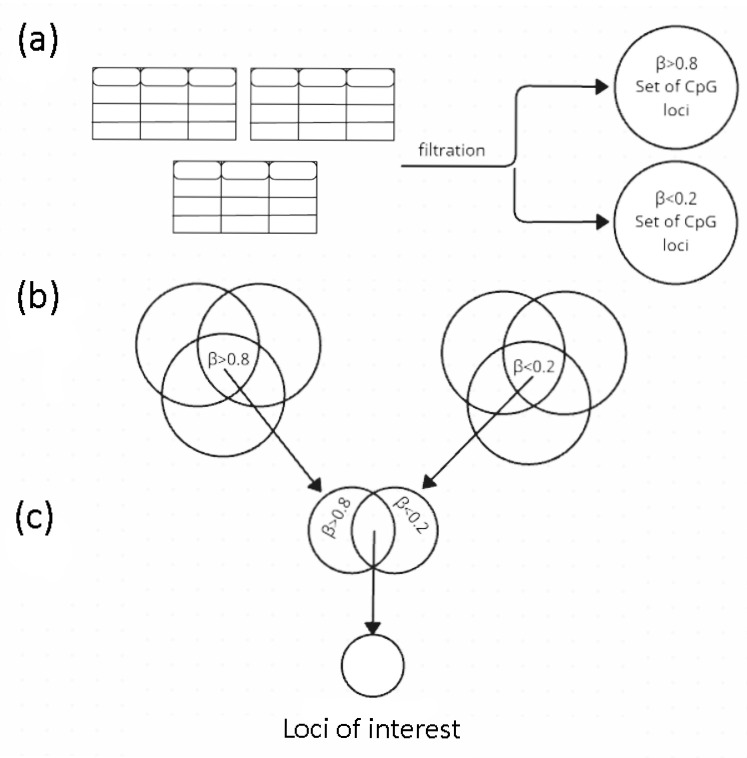
Analysis scheme: (**a**) The cleaned table of each technical repeat for each cell type (such as HUVEC, neutrophils, etc.) of each group (ECs and LCs) filtered by marginal Beta (β), and sets of CpG loci were obtained; (**b**) The sets of CpG loci with marginal Beta were intersected between cell types within each group; (**c**) The sets of CpG loci were intersected between groups of cells according to the principle of polarity of their corresponding Beta; the union of the two sets obtained in (**c**) ([ECs (β < 0.2) ∩ LCs (β > 0.8)] ∪ ([ECs (β > 0.8) ∩ LCs (β < 0.2)]) are the loci of interest. Beta—the proportion of methylated cytosines at a particular locus in a DNA sample.

Methylation microarray data on the studied samples were obtained from the open-source databases—NCBI (GEO) and EMBL-EBI (ArrayExpress): the GSE140295 dataset and the E-GEOD-39981 dataset, respectively [43,44]. The criteria for selecting these particular datasets were the following: various cell subtypes within the cell groups studied, sufficient number of biological and technical replicates, passing the quality control tests performed, and so that the number of significant Beta (at *p* < 0.05) was such that the dropped-out loci were somehow covered by the replicates and thus the number of loci available for the analysis did not decrease. Tabular data were processed using the Python (v.3.9.10) libraries: pandas (v.2.0.2), numpy (v.1.25.2), and scipy (v.1.11.4). The figures were created using the matplotlib (v.3.7.1) and seaborn (v.0.13.2) libraries. The data processing flow chart is shown in Figure 3.

First, we performed two intragroup analyses (Supplementary File S4). For ECs (LSEC, HPMEC, HCMEC, HPAEC, and HUVEC), we examine 483,032 CpGs extracted from the data obtained using Infinium HumanMethylation450 BeadChip array by Illumina [45]. For LCs (B cells, T cells (CD4+), T cells (CD8+), pan T cells, natural killers (CD16+), natural killers (CD16−), pan natural killers, neutrophils, granulocytes, monocytes, and T regulatory cells), we examined 27,578 CpGs extracted from the data obtained using Infinium HumanMethylation27 BeadChip array by Illumina [46]. Each cell type was represented by 3–6 biological replicates; each table for all replicates was filtered by the *p*-value (<0.05).

Ultimately, we analyzed 25,946 loci (the intersection of two loci sets) for intergroup comparison. Since we used data obtained from different versions of the Illumina platform, we first checked the necessity to take into consideration batch effects. To do this, we compared the lists of loci identified without and with quantile normalization (the approach was chosen due to the design of the analysis) [47] (Supplementary Figure S2) and revealed that using the original, not normalized data for the studied samples in our particular case made the analysis more rigorous (normalization expanded the list of loci (Supplementary Table S5)). Given that the goal of our study was to compare loci that we assigned to two marginal categories (close to Beta = 0, close to Beta = 1) according to empirically selected cutoffs (searching for the most extreme values), we found no necessity to use a batch effect correction method for our data analysis.

Since Beta reflects a methylation level of a CpG locus, we restricted our analysis to Beta boundaries (upper minimum and lower maximum values (Beta < min and Beta > max)) that maximize the probability of the presence/absence of a methyl mark at a given locus, to identify the most stable markers. A CpG locus that has a Beta in both cutoff intervals when performing pairwise comparisons of two cell types, is defined as ‘polar marginal’. The search for CpG loci distinguishing EC DNA from LC DNA was performed at both Beta < 0.2 and Beta > 0.8; however, to reduce the number of polar marginal CpG loci identified within cell groups, we used more stringent boundaries (Beta < 0.1 and Beta > 0.9).

## 5. Conclusions

This work offers an approach to studying endothelial dysfunction and provides data that can be used to identify the origin of DNA (due to its specific epigenetic modifications) in heterogenic vascular samples containing endothelial cells. The distinct stable methylation biomarkers identified for particular cell subtypes reveal the possibility of assessing their contribution, which, in turn, opens up additional opportunities for research in diverse areas of biomedicine, as well as clinical applications.

## Figures and Tables

**Table 1:** List of genes associated with the identified CpG loci.

Locus ^1^	Gene Name (Genomic Element)	Gene Description	GO Processes
Loci stably methylated in ECs (Beta > 0.8)			
cg25671438 ^e^	*ACAP1* (Body)	ArfGAP with coiled-coil, ankyrin repeat and PH domains 1	Regulation of GTPase activity; intracellular signaling cascade
cg06183267	*AFF3* (TSS200)	ALF transcription elongation factor 3	Transcription; development; regulation of transcription; DNA-dependent
cg07349094	*AFF3* (1stExon, 5′UTR)		
cg14451276	*AOAH* (1stExon, 5′UTR)	Acyloxyacyl hydrolase	Lipid metabolism; inflammatory response
cg15645309	*BATF* (TSS200)	Basic leucine zipper ATF-like transcription factor	Transcription; regulation of transcription; DNA-dependent; antimicrobial humoral response (sensu Vertebrata)
cg10590292	*BIN2* (Body)	Bridging integrator 2	Cell chemotaxis; phagocytosis, engulfment; plasma membrane tubulation; podosome assembly
cg08040471 *	*CYBC1* (5′UTR)	Cytochrome b-245 chaperone 1	Innate immune response; respiratory burst after phagocytosis
cg23093496	*C16orf54* (1stExon, 5′UTR)	Chromosome 16 open reading frame 54	No data
cg10037005 ^†^	*CD37* (TSS200)	CD37 molecule	Protein amino acid N-linked glycosylation
cg15046675 ^†^	*CD37* (1stExon, 5′UTR)		
cg07380416	*CD6* (5′UTR, 1stExon)	CD6 molecule	Cell adhesion; immune response
cg09902130	*CD6* (5′UTR, 1stExon)		
cg07906724	*CHRNA6* (TSS1500)	Cholinergic receptor nicotinic alpha 6 subunit	Ion transport; signal transduction; synaptic transmission
cg11804789	*CST7* (TSS200)	Cystatin F	Immune response
cg12177677	*CYTIP* (1stExon)	Cytohesin 1 interacting protein	Regulation of cell adhesion
cg21614638	*DAPP1* (TSS200)	Dual adaptor of phosphotyrosine and 3-phosphoinositides 1	Intracellular signaling cascade; protein amino acid dephosphorylation
cg19252956	*DOCK8* (Body)	Dedicator of cytokinesis 8	Cellular response to chemokine; dendritic cell migration; immunological synapse formation; memory T cell proliferation; negative regulation of T cell apoptotic process; positive regulation of establishment of T cell polarity; positive regulation of GTPase activity; positive regulation of T cell migration; regulation of Rho protein signal transduction; regulation of small GTPase-mediated signal transduction; small GTPase-mediated signal transduction
cg00661485 ^I^	*FOXI1* (1stExon)	Forkhead box I1	Transcription; development; regulation of transcription; DNA-dependent
cg18908499	*GCSAML* (TSS1500)	Germinal center-associated signaling and motility, like	Regulation of the B cell receptor signaling pathway; regulation of lymphocyte migration
cg16509569	*HEM1* (1stExon)	Homo sapiens hematopoietic protein 1	Cellular response to insulin stimulus; erythrocyte development; hemoglobin biosynthetic process; protoporphyrinogen IX biosynthetic process; response to bile acid; response to camp; response to cobalt ion; response to ethanol; response to gonadotropin; response to herbicide; response to hypoxia; response to nickel cation; response to nutrient levels; response to platinum ion; response to xenobiotic stimulus
cg16927606 ^#^	*IGFLR1* (1stExon, 5′UTR)	IGF-like family receptor 1	No data
cg17771150	*LCP1* (5′UTR)	Lymphocyte cytosolic protein 1	Actin crosslink formation; actin filament bundle assembly; actin filament network formation; animal organ regeneration; cell migration; cortical actin cytoskeleton organization; extracellular matrix disassembly; positive regulation of podosome assembly; protein kinase A signaling; regulation of intracellular protein transport; T cell activation involved in immune response
cg26158194	*LSP1* (5′UTR, 1stExon)	Lymphocyte-specific protein 1	Cell motility; signal transduction; cellular defense response
cg14519350	*OSM* (Body)	Oncostatin M	Development; immune response; cell–cell signaling; cell proliferation; regulation of cell growth; negative regulation of cell proliferation
cg05596756	*PCED1B* (5′UTR, 1stExon)	PC-esterase domain containing 1B	No data
	*PCED1B-AS1* (Body)	PCED1B antisense RNA 1	No data
cg26866325	*PPBPL2* (Body)	Pro-platelet basic protein pseudogene 2	No data
cg16890093 ^#^	*PSMB8* (TSS1500)	Proteasome 20S subunit beta 8	Proteolysis; immune response; ubiquitin-dependent protein catabolism
	*TAP1* (3′UTR)	Transporter 1, ATP-binding cassette subfamily B member	Adaptive immune response; antigen processing and presentation of endogenous peptide antigen via MHC class I; cytosol to endoplasmic reticulum transport; defense response; peptide transport; transmembrane transport
cg18384097	*PTPN7* (TSS1500, 5′UTR)	Protein tyrosine phosphatase non-receptor type 7	Protein amino acid dephosphorylation; protein amino acid dephosphorylation
cg10861751	*RGS1* (TSS200)	Regulator of G protein signaling 1	B cell activation; immune response; signal transduction; negative regulation of signal transduction; G-protein signaling; adenylate cyclase inhibiting pathway
cg24497819	*SELPLG* (TSS200)	Selectin P ligand	Cell adhesion
cg02794695	*SLA* (TSS200, Body, 5′UTR)	Src-like adaptor	Intracellular signaling cascade
	*TG* (Body)	Thyroglobulin	Hormone biosynthetic process; iodide transport; regulation of myelination; signal transduction; thyroid gland development; thyroid hormone generation
cg23612220	*TNFAIP8L2* (5′UTR)	TNF alpha induced protein 8 like 2	Innate immune response; negative regulation of inflammatory response; negative regulation of T cell activation; regulation of apoptotic process; T cell activation
cg00041575	*TSPAN32* (Body)	Tetraspanin 32	Cell–cell signaling
	*C11orf21* (TSS1500)	Chromosome 11 open reading frame 21	No data
cg13821008	*WIPF1* (5′UTR)	WAS/WASL interacting protein family member 1	Protein complex assembly; actin polymerization and/or depolymerization
Loci stably methylated in LCs (Beta > 0.8)			
cg05654164 ^#^	*C1orf52* (TSS1500)	Chromosome 1 open reading frame 52	No data
cg26143719 ^e^	*C1QTNF6* (1stExon)	C1q and TNF-related 6	Phosphate transport
cg24765079 ^#^	*CDH1* (Body)	Cadherin 1	Cell adhesion; homophilic cell adhesion
cg13354523	*CLEC1A* (5′UTR, 1stExon)	C-type lectin domain family 1 member A	Cell surface receptor signaling pathway; signal transduction
cg08578641 ^I^	*DNAI1* (Body)	Dynein axonemal intermediate chain 1	Cilium movement; determination of left/right symmetry; epithelial cilium movement involved in extracellular fluid movement; flagellated sperm motility; heart development; insulin receptor signaling pathway; outer dynein arm assembly
	*FAM219A* (TSS1500)	Family with sequence similarity 219 member A	No data
cg02293044 ^I^	*GAS2L1* (5′UTR)	Growth arrest specific 2 like 1	Cell cycle arrest
cg15475323 ^†^	*MAMSTR* (1stExon, Body, 5′UTR)	MEF2 activating motif and SAP domain-containing transcriptional regulator	Positive regulation of myotube differentiation; positive regulation of transcription by RNA polymerase II; regulation of transcription by RNA polymerase II
cg14440664	*PDCD1LG2* (TSS1500)	Programmed cell death 1 ligand 2	Immune response
cg02254407	*PLEKHB1* (TSS200, TSS1500)	Pleckstrin homology domain containing B1	Phototransduction
cg17016000	*RIN2* (TSS1500)	Ras and Rab interactor 2	Small GTPase-mediated signal transduction
cg03171924 ^I^	*RUNX3* (Body)	RUNX family transcription factor 3	transcription; cell proliferation; regulation of transcription; DNA-dependent; transcription from RNA polymerase II promoter
cg19497444 ^I^	*SLC67A1* (Body)	Solute carrier family 67 member 1	Excretion; transport; response to drug; tetracycline transport
cg04527918 ^I^	*UCN* (TSS200)	Urocortin	G-protein-coupled receptor protein signaling pathway

^1^ According to the Illumina identifier; * N-shore (the region extending up to 2 kb upstream of a CpG island); ^#^ S-shore (the region extending up to 2 kb downstream of a CpG island); ^†^ N-shelf (the region 2–4 kb upstream of a CpG island); ^e^ Enhancer; ^I^ CpG island; TSS—transcription start site.

## Data Availability

The original contributions presented in this study are included in the article. Further inquiries can be directed to the corresponding author.

## Supplementary Materials

The following supporting information can be downloaded at: https://www.mdpi.com/article/10.3390/epigenomes9040053/s1, Supplementary File S1: Statistics and annotation of the CpG loci identified by comparing Beta within each cell group; Supplementary File S2: List of CpG loci with paired polar Beta; Supplementary File S3: Statistics and annotation of the CpG loci identified by comparing Beta between the cell groups; Supplementary File S4: Annotation of the CpG loci studied; Figure S1: Biological processes involving genes associated with the CpG loci identified as distinguishing inside the cell group (ECs/LCs; Beta < 0.2 and Beta > 0.8); Figure S2: Beta distribution before and after quantile normalization; Table S1: Number of CpG loci with polar marginal Betas for each pair of cell types within the EC group; Table S2: Number of CpG loci with polar marginal Betas for each pair of cell types within the LC group; Table S3: Number of CpG loci with polar marginal Betas (at Beta < 0.2 and Beta > 0.8) for each pair of cell types between the groups; Table S4: Top 6 loci with polar marginal Betas in the LC group; Table S5: Distinct CpG loci obtained after quantile normalization.

## Abbreviations

The following abbreviations are used in this manuscript:

EC	Endothelial cell
LC	Leukocyte

## Appendix A

**Table A1:** List of genes associated with CpG loci whose methylation differs between types of endothelial cells.

Locus ^1^	Gene Name	Gene Description
cg18505593	*CCDC85C*	Coiled-coil domain containing 85C
cg05127821	*CYP26C1*	Cytochrome P450 family 26 subfamily C member 1
cg20293609	*CYR61 (CCN1)*	Cellular communication network factor 1
cg23669081	*HOXB7*	Homeobox B7
cg01128482	*HOXD3*	Homeobox D3
cg19509393	*HOXD4*	Homeobox D4
cg12195446	*IRS2*	Insulin receptor substrate 2
cg17930194	*LHX6*	LIM homeobox 6
cg08189801	*LOC404266 (HOXB-AS3)*	HOXB cluster antisense RNA 3
	*HOXB5*	Homeobox B5
cg06261059	*MCPH1*	Microcephalin 1
cg20935363	*PDE2A*	Phosphodiesterase 2A
cg26286901	*PEBP4*	Phosphatidylethanolamine binding protein 4
cg09550697	*PLEC1* *(PLEC)*	Plectin
cg19156231	*SCN8A*	Sodium voltage-gated channel alpha subunit 8
cg21974358		
cg27304952		
cg10768108	*SOX2OT (SOX2-OT)*	*SOX2* overlapping transcript
cg16276063		
cg20691722		
cg06365270	*TNXB*	Tenascin XB
cg20640374	*TRPM1*	Transient receptor potential cation channel subfamily M member 1
cg13180315	-	-
cg24145118	-	-
cg25916282	-	-
cg04115185	-	-
cg27376924	-	-
cg26962618	-	-
cg06193597	-	-
cg08426130	-	-
cg10186131	-	-
cg11114465	-	-

^1^ According to the Illumina identifier.

**Table A2:** List of genes associated with CpG loci whose methylation differs between types of leukocytes.

Locus ^1^	Gene Name	Gene Description
cg24211388	*AIF1*	Allograft inflammatory factor 1
cg12044210	*APBA2*	Amyloid beta precursor protein binding family A member 2
cg20366831	*APBA3*	Amyloid beta precursor protein binding family A member 3
cg18084554	*ARID3A*	AT-rich interaction domain 3A
cg12459502	*BCL2*	BCL2 apoptosis regulator
cg15742700	*BLK*	BLK proto-oncogene, Src family tyrosine kinase
cg08044694	*BRD4*	Bromodomain containing 4
cg09076077	*C20orf197* (*LINC02910*)	Long intergenic non-protein coding RNA 2910
cg15248035	*CCIN*	Calicin
cg14102807	*CD19*	CD19 molecule
cg11939496	*CD244*	CD244 molecule
cg24841244	*CD3D*	CD3 delta subunit of the T cell receptor complex
cg15880738	*CD3G*	CD3 gamma subunit of the T cell receptor complex
cg22512531	*CRTAM*	Cytotoxic and regulatory T cell molecule
cg07285167	*CSF3R*	Colony-stimulating factor 3 receptor
cg09971811	*CST7*	Cystatin F
cg24777950	*CTSG*	Cathepsin G
cg16280667	*CXCR5*	C-X-C motif chemokine receptor 5
cg22381196	*DHODH*	Dihydroorotate dehydrogenase (quinone)
cg04451770	*ENTPD1*	Ectonucleoside triphosphate diphosphohydrolase 1
cg11024597	*ECRG4*	ECRG4 augurin precursor
cg00415993	*F2RL2*	Coagulation factor II thrombin receptor-like 2
	*IQGAP2*	IQ motif containing GTPase-activating protein 2
cg14654385	*FERMT3*	FERM domain-containing kindlin 3
cg00226923	*FGD2*	FYVE, RhoGEF, and PH domain-containing 2
cg11254522	*FGR*	FGR proto-oncogene, Src family tyrosine kinase
cg27461196	*FXYD1*	FXYD domain-containing ion transport regulator 1
cg13703437	*FYB* (*FYB1*)	FYN binding protein 1
cg27606341		
cg12125117	*GPR97* (*ADGRG3*)	Adhesion G protein-coupled receptor G3
cg17356733	*IFNGR2*	Interferon gamma receptor 2
cg12380764	*IL19*	Interleukin 19
cg02656594	*IL21R*	Interleukin 21 receptor
cg26757673	*IL2RB*	Interleukin 2 receptor subunit beta
cg03801286	*KCNE1*	Potassium voltage-gated channel subfamily E regulatory subunit 1
cg23090046	*KLC1*	Kinesin light chain 1
cg01500140	*LIM2*	Lens intrinsic membrane protein 2
cg00546897	*LOC284837* (*AATBC*)	Apoptosis-associated transcript in bladder cancer
cg16097772	*LYZ*	Lysozyme
cg10126923	*NKG7*	Natural killer cell granule protein 7
cg21991396	*NLRP3*	NLR family pyrin domain-containing 3
cg02863947	*NR1I2*	Nuclear receptor subfamily 1 group I member 2
cg00795812	*PDCD1*	Programmed cell death 1
cg13650156	*PILRA*	Paired immunoglobulin-like type 2 receptor alpha
cg20748065	*POR*	Cytochrome p450 oxidoreductase
cg14182690	*RUNX3*	RUNX family transcription factor 3
cg03330678	*SEPT9* (*SEPTIN9*)	Septin 9
cg22242539	*SERPINF1*	Serpin family F member 1
cg08458487	*SFTPD*	Surfactant protein D
cg18854666	*SLC11A1*	Solute carrier family 11 member 1
cg11283860	*SLC45A1*	Solute carrier family 45 member 1
cg14127336	*TCL1A*	TCL1 family AKT coactivator A
cg16177830	*TNFRSF17*	TNF receptor superfamily member 17
cg06812844	*TRPM2*	Transient receptor potential cation channel subfamily M member 2
cg17496921	*TSPAN16*	Tetraspanin 16
cg02600394	*TXK*	TXK tyrosine kinase
cg24091474	*TYROBP*	Transmembrane immune signaling adaptor TYROBP
cg00134539	*UBASH3A*	Ubiquitin-associated and SH3 domain-containing A

^1^ According to the Illumina identifier.
