# Supplementary material for: Epigenome-Wide Search for Distinctive Methylation Biomarkers of Endothelial and Leukocyte DNA
Source: Epigenomes. 2025 Dec 17;9(4):53. doi: 10.3390/epigenomes9040053 (PMC12731890; doi:10.3390/epigenomes9040053)
Supplement: Supplementary file 1 [file epigenomes-09-00053-s001.zip › Supplementary.pdf]

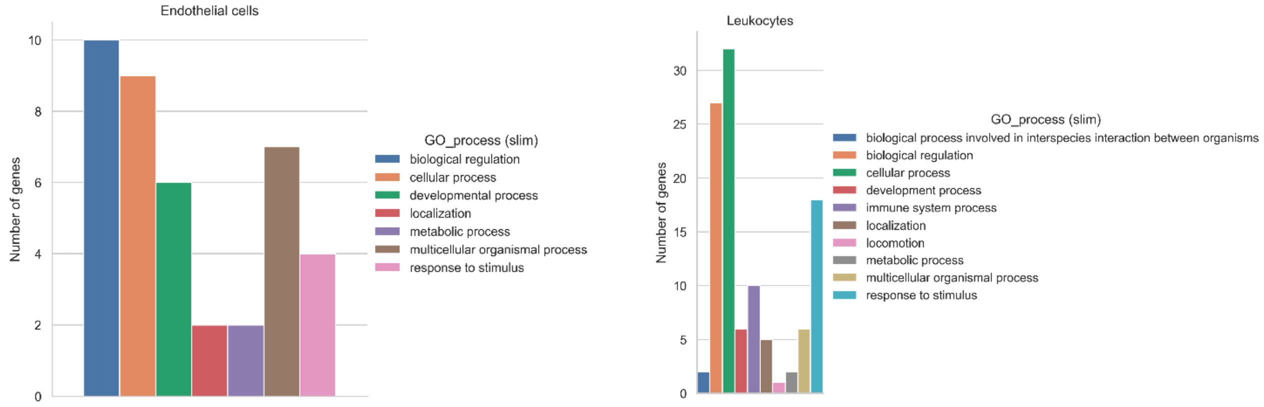

Figure S1. Biological processes involving genes associated with the loci identified as distinguishing inside the cell group (ECs / LCs; Beta < 0.2 and Beta > 0.8).

Table S1. Number of CpG loci with polar marginal Betas for each pair of cell types within the EC group (Beta < 0.2 and Beta > 0.8).

*	HCMEC	HPAEC	HPMEC	HUVEC	LSEC
**HCMEC**	0				
**HPAEC**	15	0			
**HPMEC**	19	8	0		
**HUVEC**	279	170	105	0	
**LSEC**	20	122	5	319	0

* HCMEC – human cardiac microvascular endothelial cells; HPAEC – human pulmonary artery endothelial cells; HPMEC – human pulmonary microvascular endothelial cells; HUVEC – human umbilical vein endothelial cells; LSEC – liver sinusoidal endothelial cell.

Table S2. Number of CpG loci with polar marginal Betas for each pair of cell types within the LC group (Beta < 0.2 and Beta > 0.8).

*	B-cells	Pan T-cells	T-cells (CD4+)	T-cells (CD8+)	Tregs	Pan NK	NK (CD16+)	NK (CD16-)	Granulo cytes	Neutro phils	Mono cytes
**B-cells**	0										
**Pan T-cells**	34	0									
**T-cells (CD4+)**	40	0	0								
**T-cells (CD8+)**	48	0	0	0							
**Tregs**	35	0	0	1	0						
**Pan NK**	1	7	11	5	8	0					
**NK (CD16+)**	49	13	27	7	22	0	0				
**NK (CD16-)**	25	10	12	14	16	0	1	0			
**Granulocytes**	82	103	116	120	104	62	94	78	0		
**Neutrophils**	75	97	109	109	97	56	87	71	0	0	
**Monocytes**	63	100	105	119	105	52	87	64	11	10	0

* NK – natural killer cells; Tregs – regulatory T-cells; CD – cluster of differentiation.

Table S3. Number of CpG loci with polar marginal Betas (Beta < 0.2 and Beta > 0.8) for each pair of cell types between the groups.

*	B-cells	Pan T-cells	T-cells (CD4+)	T-cells (CD8+)	Tregs	Pan NK	NK (CD16+)	NK (CD16-)	Granulo cytes	Neutro phils	Mono cytes
**HCMEC**	108	97	102	104	100	84	117	88	166	155	154
**HPAEC**	157	133	136	146	138	127	162	129	233	218	220
**HPMEC**	120	103	104	112	108	94	127	96	178	163	173
**HUVEC**	192	170	178	179	176	161	198	163	281	266	270
**LSEC**	122	109	114	119	115	97	140	103	183	170	174

* HCMEC – human cardiac microvascular endothelial cells; HPAEC – human pulmonary artery endothelial cells; HPMEC – human pulmonary microvascular endothelial cells; HUVEC – human umbilical vein endothelial cells; LSEC – liver sinusoidal endothelial cell; NK – natural killer cells; Tregs – regulatory T-cells; CD – cluster of differentiation.

Table S4. Top 6 loci with polar marginal Betas in the LC group.

*	B-cells	Pan T-cells	T-cells (CD4+)	T-cells (CD8+)	Tregs	Pan NK	NK (CD16+)	NK (CD16-)	Granulo cytes	Neutro phils	Mono cytes
**cg17356733**	↑**	↑	↑	-‡	↑	↑	↑	↑	↓†	↓	↓
**cg18084554**	↑	↑	↑	↑	-	-	-	↑	↓	↓	↓
**cg20748065**	-	↑	↑	↑	↑	-	↑	↑	↓	↓	↓
**cg21991396**	↑	↑	↑	↑	↑	↑	↑	-	↓	↓	↓
**cg22381196**	↑	↑	↑	↑	↑	↑	↑	↑	↓	↓	↓
**cg27606341**	-	↑	↑	↑	↑	-	↑	-	↓	↓	↓

* NK – natural killer cells; Tregs – regulatory T-cells; CD – cluster of differentiation; ** Loci with Beta>0.9; † Loci with Beta<0.1; ‡ Out of range (Beta < 0.1 and Beta > 0.9).

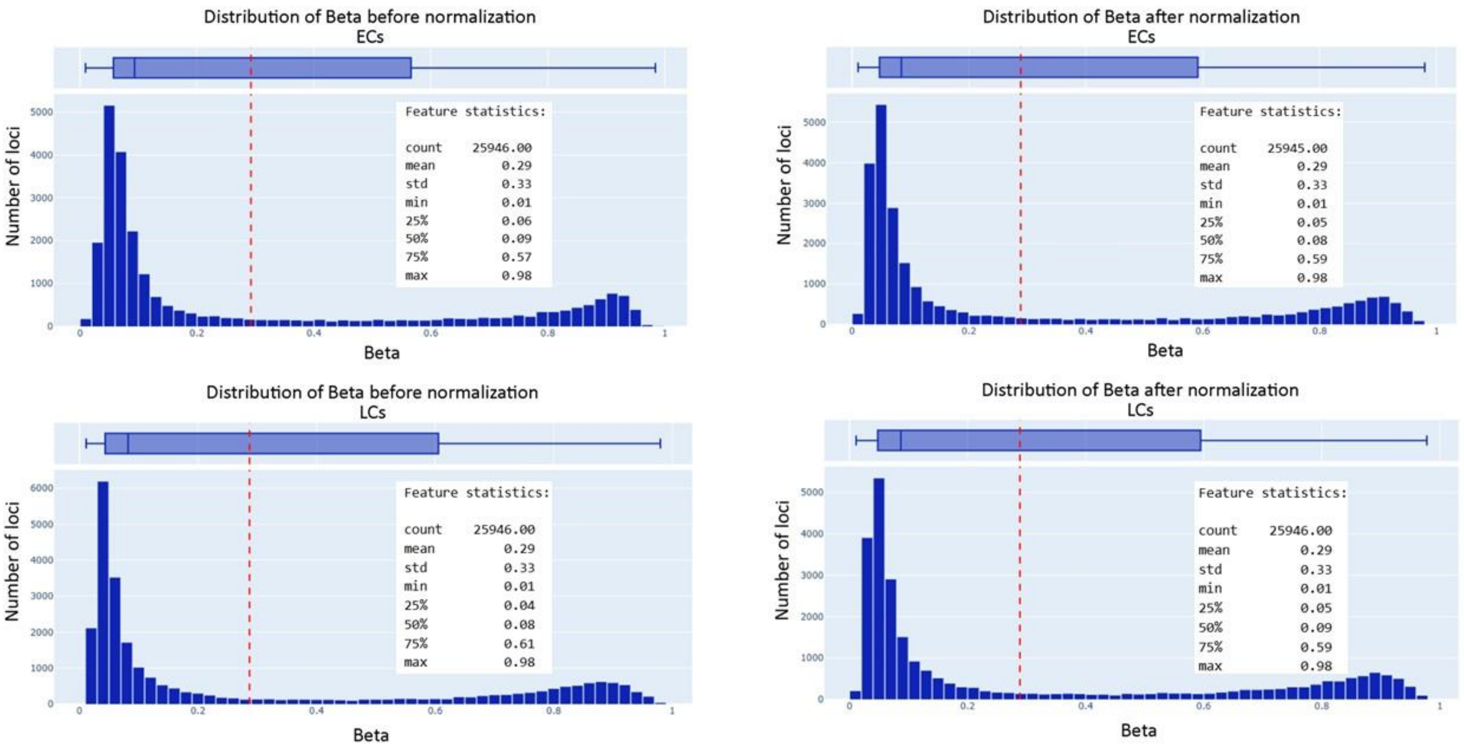

Figure S2. Beta distribution before and after quantile normalization. For simplicity, the average Betas for all endothelial cells (ECs) and leukocytes (LCs) studied are shown. The red dotted line designates the average value.

Table S5. Distinct CpG loci obtained after quantile normalization.

CpG locus	ECs	LCs
**Included loci after normalization**		
cg16776350	0.84	0.10
cg05861567	0.83	0.10
cg09196959	0.83	0.10
cg27631256	0.85	0.10
cg26151675	0.83	0.07
cg22325572	0.84	0.07
cg04915566	0.86	0.10
**Excluded loci after normalization**		
cg15645309	0.87	0.15[1]
cg26866325	0.89	0.18[2]
cg08040471	0.91	0.07[3]
cg19252956	0.91	0.13[4]

[1] Average Beta (Monocytes)=0.21, average Beta (Neutrophils) = 0.21 (reason for exclusion);

[2] Average Beta (Granulocytes) = 0.24, average Beta (Monocytes) = 0.24 (reason for exclusion);

[3] Average Beta (NK (CD16-)) = 0.21 (reason for exclusion);

[4] Average Beta (Granulocytes) = 0.21 (reason for exclusion).
